# Modelling Growth and Form of the Scleractinian Coral *Pocillopora verrucosa* and the Influence of Hydrodynamics

**DOI:** 10.1371/journal.pcbi.1002849

**Published:** 2013-01-10

**Authors:** Nol Chindapol, Jaap A. Kaandorp, Carolina Cronemberger, Tali Mass, Amatzia Genin

**Affiliations:** 1 Section Computational Science, University of Amsterdam, Amsterdam, The Netherlands; 2 Institute of Marine and Coastal Sciences, Rutgers University, New Brunswick, New Jersey, United States of America; 3 Interuniversity Institute for Marine Sciences, Eilat, Israel; 4 Institute of Life Sciences, Hebrew University of Jerusalem, Jerusalem, Israel; University of Auckland, New Zealand

## Abstract

The growth of scleractinian corals is strongly influenced by the effect of water motion. Corals are known to have a high level of phenotypic variation and exhibit a diverse range of growth forms, which often contain a high level of geometric complexity. Due to their complex shape, simulation models represent an important option to complement experimental studies of growth and flow. In this work, we analyzed the impact of flow on coral's morphology by an accretive growth model coupled with advection-diffusion equations. We performed simulations under no-flow and uni-directional flow setup with the *Reynolds* number constant. The relevant importance of diffusion to advection was investigated by varying the diffusion coefficient, rather than the flow speed in *Péclet* number. The flow and transport equations were coupled and solved using COMSOL Multiphysics. We then compared the simulated morphologies with a series of Computed Tomography (CT) scans of scleractinian corals *Pocillopora verrucosa* exposed to various flow conditions in the *in situ* controlled flume setup. As a result, we found a similar trend associated with the increasing *Péclet* for both simulated forms and *in situ* corals; that is uni-directional current tends to facilitate asymmetrical growth response resulting in colonies with branches predominantly developed in the upstream direction. A closer look at the morphological traits yielded an interesting property about colony symmetry and plasticity induced by uni-directional flow. Both simulated and *in situ* corals exhibit a tendency where the degree of symmetry decreases and compactification increases in conjunction with the augmented *Péclet* thus indicates the significant importance of hydrodynamics.

## Author Summary

A long-standing question in marine biology and coral biology is the morphological plasticity of corals, sponges and other marine sessile organisms and the influence of water movement. Usually branching species tend to develop symmetrical colonies where branches are being formed in all directions. There is a long standing discussion if this process in which colonies develop symmetrical colonies is controlled by genes or by the environment. In this work, we address this question for the scleractinian coral *Pocillopora verrucosa*. We first have acquired coral colonies from a controlled *in-situ* flow experiment where the coral was growing under uni-directional flow conditions. The corals colonies were scanned using a Computed Tomography (CT) technique used for medical imaging and industrial imaging. We have developed a simulation for the growth and form of corals and the influence of water movement. We have compared the simulated morphologies to the three dimensional images obtained with the CT scanner. We have found that coral's branches predominantly develop in the upstream part of the colony and an asymmetrical colony is being formed under uni-directional flow conditions. Our results confirm that growth of the coral is strongly influenced by the flow conditions.

## Introduction

Some corals are known to have a high degree of morphological plasticity along different environmental conditions [1]–[3]. Light and water motion are the main environmental factors related to morphological variations of a coral colony [2]. The water movement over a reef is responsible to transport the necessarily nutrients [4]–[6], to flush the waste products [7], increase particle capture [8], and enhance photosynthesis and respiration [9]–.

There is evidence to suggest that different morphologies emerge in response to changes in hydrodynamic energy [12], [13]. In particular for branching corals *Pocillopora damicornis*, *Seriatopora hysterix* and the hydro coral *Millepora alcicornis*, measurements showed that their morphology had changed in accordance with the intensity of the ambient flow – a compact shape under high flow environment, and a more open growth form with thin branches under lower flow [13]. Earlier experiments with branching corals in flume studies [14] suggested that densely packed branching colonies act as a solid body. Water flow (even for a relatively high flow velocity about 20 cm s^−1^) starts to circumvent the colony creating a stagnant region inside. In a low energy environment, sparely spacing branching structures allow flow to penetrate deeper inside the colony as opposed to densely compacted branching. Chang and collaborators [15] reported a variation in intra-colony flow pattern in scale models of *Stylophora pistillata*. Colonies from the low flow environment were reported to distribute the flow velocity more evenly throughout the interior compared to the colony collected from higher flow regime. Reidenbach et al. [16] estimated mass transfer in unidirectional and oscillatory flows of three coral species - *Stylophora pistillata*, *Pocillopora verrucosa and Pocillopora compressa*. Mass transfer rate was shown to be a function of the physical flow characteristic and the morphology of branching structure. For unidirectional current, mass transfer rate of sparsely branched corals was observed to be larger for increasing flow velocity. At a higher frequency oscillatory flow, however, the compactness in branch spacing greatly enhance mass transfer rate through the inner structure of the branches.

Water motion also influences the coral's physiological mechanisms during photosynthesis and respiration [10], [17]. At the tissue level epithelial transport of ions, Dissolved Inorganic Carbon (DIC) and oxygen occurs by passive diffusion due to a standing gradient of these compounds around the coral branches. These gradients, created in the Diffusive Boundary Layer (DBL), are directly influenced by the effect of flow [4], [18].

**Figure 1:**
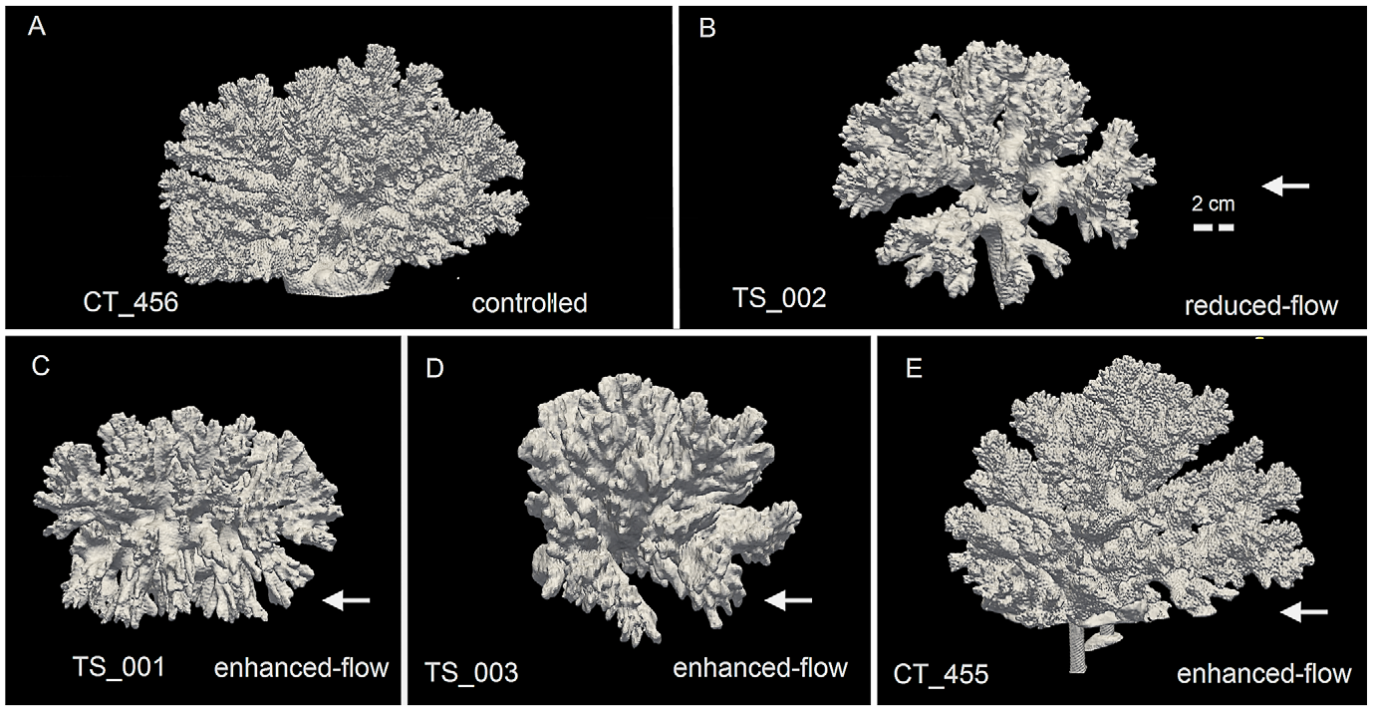
Computed Tomography (CT) scans of scleractinian coral *Pocillopora verrucosa*. (A) A controlled coral exposed to ambient current with the average near-bottom velocity of 5 cm s^−1^ [46]. (B) A coral that was grown in an *in situ* controlled flume setup with “reduced flow” condition (∼1 cm s^−1^) (C–E) Corals were grown in the *in-situ* controlled flumes setup with “enhanced flow” condition (15–20 cm s^−1^) [32]. Arrows indicate flow direction. The labels of the CT scans are located at the bottom of each figure (see Table 1).

In two studies by Nakamura et al. [19], [20] the susceptibility of the coral *Acropora digitifera* to bleaching during hot periods and under low flow conditions was demonstrated. Another recent study [11] focused on the effect of flow-dependent efflux of oxygen during photosynthesis, showing the importance of water movement to remove the excessive oxygen from the organism, as oxygen accumulation inhibits photosynthesis. Under natural conditions branching corals (e.g. *Pocillopora sp.*, *Stylophora pistillata*, *Madracis mirabilis*) tend to develop symmetrical branching colonies - as shown in Figure 1A. Two hypotheses can be found in the literature to explain this phenomenon. According to some authors gene regulation is controlling the symmetrical shape [21]–[23]; whereas an alternative explanation is that the symmetry of the colony can be explained by environmental influences and the supply of nutrients [24]. In order to investigate these hypotheses, Mass and Genin [25] conducted an in-situ control flow study. The authors observed that *Pocillopora verrucosa* growing in unidirectional flow condition developed an asymmetrical branching form with strong growth in the upstream direction of flow leading to the conclusion that extrinsic factors control the colony symmetry [11], [25]. These results show that environmental factors contribute greatly to the asymmetry in coral colonies.

Former simulation models were used to address the influence of flow on the morphology of scleractinian corals. The first approach used a three-dimensional aggregation model (represented on a cubic lattice) coupled with a hydrodynamic model [26]. However a major problem in this model was that the morphology of three-dimensional aggregates on a cubic lattice was very artificial and almost impossible to compare with the morphology of a branching coral. Subsequent work [27] used an accretive growth model in which the branching mechanism of the coral was controlled by local curvatures. In both approaches the advection-diffusion equations were solved using the lattice Boltzmann method combined with the momentum propagation method [28]. Still, a major issue with the latter method was to become unstable for relatively very low flow velocities.

In three more recent papers [24], [29], [30], an improved version of the accretive growth model has been used - the polyp-based accretive growth model, in which branching is no longer controlled by a branching rule but it is an emergent phenomenon of the model and is caused by local differences in simulated nutrient concentrations at the surface. With this enhanced accretive growth model combined with the lattice Boltzmann method and the momentum propagation method it was possible to compare simulated morphologies to real coral morphologies. Because of the numerical instabilities, only morphologies in the diffusion-limited range have been simulated so far. In the present work we use the polyp-based accretive growth model to simulate coral morphologies and we are, for the first time, able to increase the flow speed up to 5 cm/s by using the advection-diffusion solver from COMSOL Multiphysiscs [31].

In order to study the emergence of symmetrical and asymmetrical growth forms under different hydrodynamic conditions, we used a previously published computational growth model [29] coupled with a model of nutrients transport through flow [31]. We computed local concentrations of “nutrients” around a simulated object, where the growth rate is driven by the absorbed quantities of simulated nutrients. The improved accretive growth model generated a number of flow-induced growth forms, depending on the model parameters. The simulated growth forms were compared to Computer Tomography (CT) scans of real corals exposed to different flow conditions in a controlled flume setup [25], [32] while changes in symmetry and compactification were evaluated with regard to corresponding changes in *Péclet* numbers

## Materials and Methods

### Data acquisition

The experimental data used in this work originated from samples of *Pocillopora verrucosa* colonies collected after the end of two flume tank experiments conducted at H. Steinitz Marine Biology Laboratory, Eilat, Israel (Red Sea, 29°30′N, 34°56′E) [25], [32]. Both experiments took place in an *in situ* controlled flow environment. The first two samples (Figure 1C and 1E) were scanned using CT scanning techniques [27] with a voxel spacing of 0.35 mm × 0.35 mm × 0.30 mm and 262 to 378 numbers of slices per scan, depending on the colony size. The remaining samples (Figure 1 A,B,D) were scanned with a voxel spacing of 0.59 mm×0.59 mm×0.40 mm. Visualization of the CT scans was carried out with Paraview [33].

### Flow driven accretive growth model

**Figure 2:**
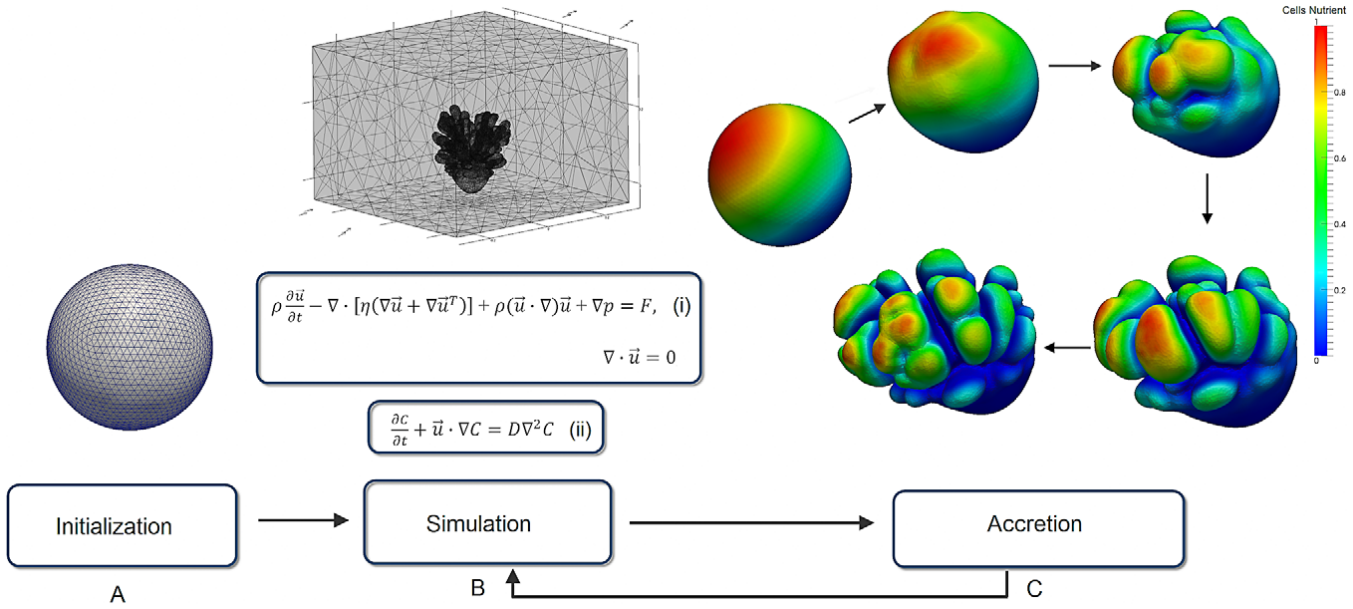
Schematic diagram of the simulation. (A) A spherical object represents an initial growth state of the simulation (first growth step) (B) A simulation phase involves solving the Navier-Stokes equations (i) and the advection-diffusion equation (ii). (C) Accretion phase translocates absorbed nutrients from previous simulation phase to a new growth layer hence, after a few consecutive growth steps, spontaneous branching occurs.

**Figure 3:**
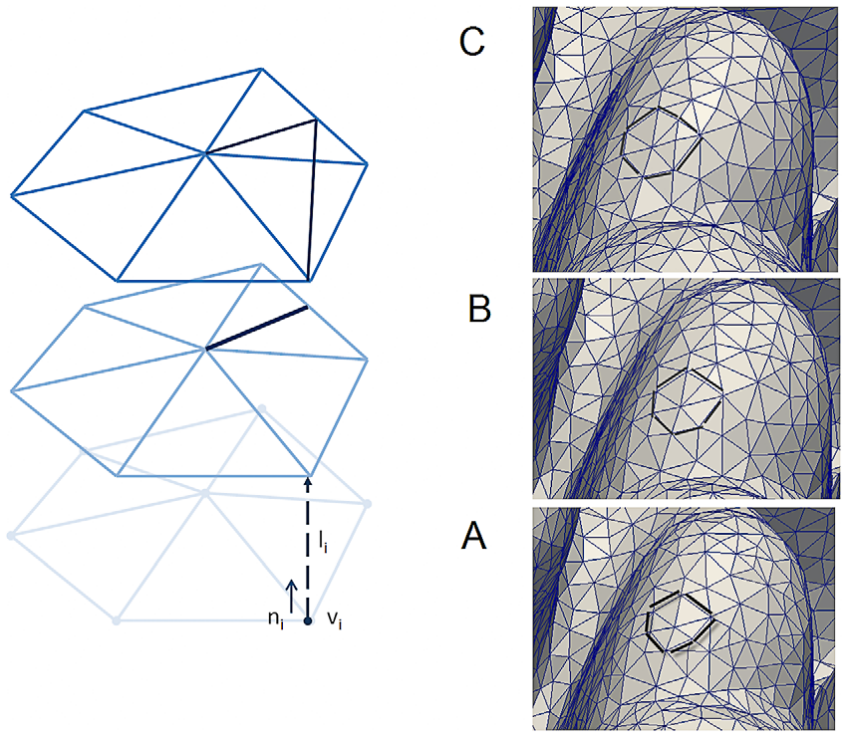
An example of three consecutive accretive growth steps. (A–C) Accretive growth steps; vertex v_i_ represents a simulated corallite. The new layer is constructed along the direction of normal vector n_i_ of the vertex v_i_. A, B and C are three consecutive growth steps where triangles are inserted once the surface of the object increases.

The simulations in this study are based on the accretive growth model [29]. The growth process is initialized with a triangulated spherical object (Figure 2A). Each vertex of the triangle represents a polyp which is interconnected with other simulated polyps. The growth of the simulated polyps is constructed along the direction of normal vector n_i_ of the vertices v_i_ (Figure 3). The development of the simulated coral can be viewed as an accretive process of the evolving interface consisted of layers constructed on top of each other (see Figures 2C and 3). The growth of simulated corals is assumed to be locally limited by the availability of nutrients (e.g. Dissolved Inorganic Carbon) from the surrounding water. Simulated nutrients are absorbed at the surface nodes and can be translocated to their neighbor by a surface diffusion process.

**Figure 4:**
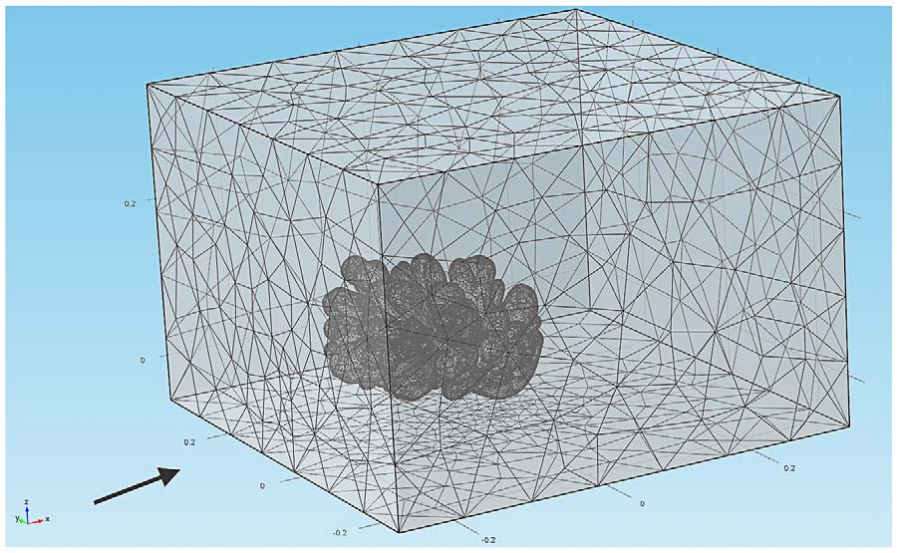
Finite element meshes representation of the environment around the simulated coral. The arrow indicates the direction of the flow.

The simulation domain consists of two compartments: a rectangular channel (60 cm length × 60 cm width × 40 cm height), where fluid is supposed to enter and exit the domain from left to right. The simulation is initialized with a triangulated spherical object with a diameter of 6 cm representing the (initial) simulated coral. The discretization of the simulation domain is done using the Galerkin finite element method in COMSOL Multiphysics [31] Figure 4 demonstrates an example of the finite element mesh from the discretization of the simulation domain. The flow field 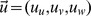 is given by the incompressible Navier-Stokes (NVS) equations and the continuity equation
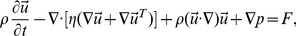
(1)

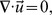
where *F* is the external volume force, which was set to 0, *u* is the fluid velocity, *p* is the pressure, 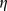 is the dynamic viscosity and 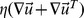 is the stress tensor that results from the fluid viscosity. Fluid entered the simulation domain with the initial velocity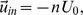 where 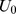 is the uniform inlet velocity profile along the normal direction of the entry point and was set to 0.05 m s^−1^. The boundary conditions of the substratum and surface of the simulated corals were set to the no-slip condition where velocity vector 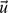 was zero. At the outlet the pressure and viscous stress is set to zero 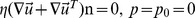. This boundary specifies vanishing viscous stress along with a Dirichlet condition on the pressure. In general, this perscription is numerically stable and admits total control of the pressure level along the entire boundary if the viscous stresses downstream are homogenous [34]. The rest of the boundaries were open boundaries with no viscous stress 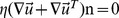. This condition describes boundaries that are open to large volumes of fluid, which is free to enter or leave the simulation domain.

The impact of the flow is described using the non-dimensional Reynolds number, defined as
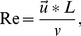
(2)in which *L* is the characteristic length (m), 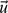 is the mean velocity of the fluid (m s^−1^), 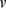 is the kinematic viscosity (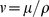), 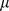 is the dynamic viscosity of the fluid (Pa s) and 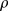 is fluid density (kg m^−2^). Assume that coral branches can be roughly approximated by a circular cylinder. Depending on the *Re* number, the wake behind a circular cylinder behaves differently from steady larminar flow (*Re*<40) to unsteady with periodic behavior (40<*Re*<200). The wake becomes unstable for *Re*>200 and completely turbulent for *Re*>5000. For the dynamic viscosity 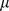 = 1.0 e^−3^ Pa s and u∼0.05 m s^−1^, for a 2 cm branch (L∼0.02 m), we will have *Re_branch*∼1000. If the whole colony is assumed to be a circular cylinder, *Re_colony* will be ∼5000. From this estimation, the flow through coral branches will become turbulent thus different eddies of different sizes and strengths can be expected along the wake [16]. This gives rise to instabilities in the Navier-Stokes equations. In our simulations turbulence is suppressed by increasing the viscous forces over inertial forces (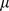 = 5.0 e^−2^ Pa s). Augmenting fluid viscosity 50 times more than water, the *Re_branch* and *Re_colony* are greatly reduced (*Re_branch*∼20, *Re_colony*∼100) resulting in a laminar streamline flow thus allows reasonable time to find the steady-state solutions. For the analysis of stationary time independent solutions, we use a nonliner solver with an iterative method to solve the linearsized equations. The stationary solutions were calculated with the biconjugate gradient stabilized (BiCGStab) linear system solver and Vanka preconditioner from COMSOL Multiphysics [31]. Vanka update was done two times in the preconditioner iteration and solved using generalized minimal residual (GMRES) method. The relative tolerance of the iterative solver is set to 1.0 e^−3^. The initial velocity 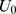 was used only in the first simulation step whereas in the subsequent simulations, the solutions from the previous time step were reused as the new initial conditions. This makes the solution converge faster due to small changes in geometry for each simulation of the growth step. In the supplementary Text S1, we provide a MATLAB script describing COMSOL routines (v3.5a) and parameter settings that were used for our advection-diffusion simulations.

After the solutions of NVS equations had been found, simulated nutrient entered the simulation domain from all sides and were absorbed by the simulated coral. Subsequently, the amount of absorbed nutrients were determined by solving the advection-diffusion equation,
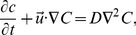
(3)where *c* is the concentration gradient, 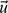 is the velocity vector obtained from the solution of the NVS equations and *D* is the diffusion coefficient. The concentration of nutrients was initialized with the idealized value of 1.0 mol m^−3^ on all boundaries except the substratum and the simulated object, which were set to 0. The stationary time independent solutions were computed using the BiCGStab linear System Solver and Vanka preconditioner [31]. After the relative tolerance of the iterative solver had reached a stopping criterion of 1.0e^−6^, the absorbed flux at each of the vertex was outputted by interpolating the concentration of nutrient at a small distance (*l*) along the normal direction of the vertices. Since the concentration on the coral's surface is zero, we normalize the absorbed flux by the maximum concentration *c_max_* 
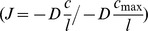
, which results in a flux that is independent of *D* and *l* and has a maximum of a unit concentration 1 mol m^−3^.

These absorbed quantities were translocated to the neighboring vertices by means of surface diffusion,
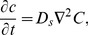
(4)where 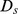 is the surface diffusion coefficient. The surface diffusion process can be interpreted as a simplified mean to translocate energy supply, in terms of absorbed concentration in this case, between adjacent regions on the simulated object's surface. Diffusivity on the surface is controlled by 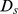, slower 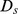 leads to minimun translocation and induces a small branching pattern, whereas faster 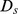 facilitates bulky branches due to scattered resources on the surface (Filatov et al. [30]). Since the branching pattern is sensitive to the variation of surface diffusivity, to address the impact of flow, we kept 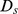 constant to a value of 3.0e^−4^ m^2^ s^−1^ in all of the simulations – this value induces intermediate branching pattern.

After calculating surface diffusion, the translocated concentrations of nutrients at each vertex were used to determine the thickness of a new growth layer.

For a vertex *v_i_*, the length of the newly extended growth vector *l_i_* along the normal direction of *v_i_* is regulated by the growth function:
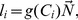
(5)where *g*(*C_i_*) is a nutrient response function that obey saturation kinetics, which converts the translocated nutrients *C_i_* at *v_i_* into the local thickness *l_i_* of the new growth layer. In order to relate the growth rate to the translocated nutrient, it is assumed that the growth rate approaches a saturation state at high nutrient uptake thus limiting the extension of the local thickness 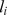. This type of growth function is a sigmoid function:
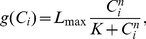
(6)where *L_max_* is an asymptotic maximum growth rate, 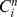 is the translocated nutrient at a vertex *v_i_*. The exponent *n* defines the kinetic order of the growth rate with respect to *C_i_*. The model parameters 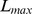 and *K* denote the characteristic growth curve of the growth function. For every growth simulation, *L_max_* was set to 1.0 e^−3^ m and *K* was set to 1 mol m^−3^. This setup corresponds to an extension rate approximately about half a millimeter because 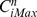 is normalized to 1 mol m^−3^. Since the growth rate of a coral colony is approximately about 1–2 cm per year, the size of the simulated object after a consecutive run of 150 growth simulations (∼10 cm) is comparable to the size of the speciements collected fron the *in situ* experiments (∼10 cm). The last parameter exponent *n* was increased to *n* = 1.2 to yield a slightly steeper growth curve. In the appendix we demonstrate the influence of the parameter *n* on the simulated morphologies (Figure S2).

To compare the influence of hydrodynamics to the morphology obtained from the simulations and CT-scanned corals, we used the *Péclet* number (*Pe*). In the absence of fluid motion diffusion dominates transport and results in a lower *Pe*, while large values of *Pe* denote a regime where advection dominates.
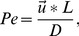
(7)where 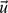 is the mean flow velocity and *L* is the characteristic length, and *D* is the diffusion coefficient. For each simulation, the impact of flow over diffusion was simulated by gradually decreasing the diffusion coefficient *D* in Equation 3 by a factor of 10 from 1.0 m^2^ s^−1^ to 1.0e^−5^ m^2^ s^−1^. The variation allowed us to study the resulting morphology by keeping 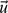 constant. The characteristic length *L* used here was the average diameter of the terminal branches of the simulated coral (*dc*). This parameter can be considered as a morphological invariant property of a branching object [35]. The list of the parameters we used in the simulations can be found in Table 1.

**Table 1:** Parameters used for simulations and parameters used to calculate *Pe* number, surface/volume ratio and symmetry magnitude.

Figure/Label	Velocity (m s^−1^)	Dynamic Viscosity (Pa s)	Fluid Density (kg m^−3^)	Diffusion Coefficient (m^2^ s^−1^)	Surface Diffusion (m^2^ s^−1^)	dc (m)	*Pe branch*	*Re branch*	Surface area (m^2^)	Volume (m^3^)	Surface Volume Ratio (m^−1^)	Sm_ mag_mean_ (m)
6A/SIM_NO_FLOW	0	N/A	N/A	1	3.00e^−4^	2.42e^−3^	0	0	4.95e^−2^	1.19e^−4^	416	3.27e^−3^
6B/SIM_FLOW_D1	0.05	5.00e^−2^	1.00e^3^	1.00e^−1^	3.00e^−4^	2.26e^−3^	1.13e^−3^	2.26	4.59e^−2^	1.12e^−4^	410	3.98e^−3^
6C/SIM_FLOW_D2	0.05	5.00e^−2^	1.00e^3^	1.00e^−2^	3. 00e^−4^	2.10e^−3^	1.05e^−2^	2.10	3.64e^−2^	8.98e^−5^	405	11.25e^−3^
6D/SIM_FLOW_D3	0.05	5.00e^−2^	1.00e^3^	1.00e^−3^	3. 00e^−4^	1.94e^−3^	9.7e^−2^	1.9	3.22e^−2^	7.74e^−5^	416	28.77e^−3^
6E/SIM_FLOW_D4	0.05	5.00e^−2^	1.00e^3^	1.00e^−4^	3.00e^−4^	2.26e^−3^	1.13	2.26	2.30e^−2^	6.34e^−5^	363	33.45e^−3^
6F/SIM_FLOW_D5	0.05	5.00e^−2^	1.00e^3^	1.00e^−5^	3.00e^−4^	N/A	∼11	∼2	3.45e^−2^	1.03e^−4^	335	N/A
1A/CT_456	0.05	1.00e^−3^	1.00e^3^	1.00e^−3^	N/A	2.71e^−3^	1.36e^−1^	135.5	1.32e^−1^	1.53e^−4^	863	2.4e^−3^
1B/TS_002	0.01	1.00e^−3^	1.00e^3^	1.00e^−3^	N/A	1.63e^−3^	1.63e^−2^	16.3	4.97e−^2^	8.46e^−5^	587	1.93e^−3^
1C/TS_001	0.15	1.00e^−3^	1.00e^3^	1.00e^−3^	N/A	1.25e^−3^	1.88e^−1^	187.5	8.64e^−2^	1.28e^−4^	675	3.96e^−3^
1D/TS_003	0.15	1.00e^−3^	1.00e^3^	1.00e^−3^	N/A	1.51e^−3^	2.27e^−1^	226.5	4.13e^−2^	7.15e^−5^	578	7.36e^−3^
1E/CT_455	0.15	1.00e^−3^	1.00e^3^	1.00e^−3^	N/A	1.92e^−3^	2.88e^−1^	288	1.03e^−1^	1.38e^−4^	746	20.53e^−3^

SIM_× are simulated objects and CT_× and TS_× are Computer Tomography scans of *Pocillopora verrucosa*.

### Estimation of *Pe* number in real corals

While for simulated objects exact *Pe* numbers were calculated, the value of *Pe* for the real corals could only be approximated. Here we used the characteristic velocity acquired from the flume experiment [32]. The characteristic velocity in which the “reduced-flow coral” (Figure 1B) and the “enhanced-flow coral” (Figure 1C–E) were grown was assumed to be 1.0e^−2^ cm s^−1^ and 15 cm s^−1^ respectively. The diffusion coefficient was assumed to be 1.0e^−3^ m^2^ s ^−1^ and invariant for the whole estimation of *Pe* number. The calculation of characteristic length *L* was the same as the length scale calculated from the simulation, which was the average diameter of the terminal branches (*dc* in Table 1).

### Morphometric analysis

Due to the complexity of coral's geometry, the morphological analysis required an alternative approach from traditional landmark-based morphometrics [36], which is more suitable for unitary organisms. In our work, we used a morphometric method that extracted a number of localized variables such as terminal branch thickness (*dc*) and local direction of growth of the branching tips [35]. These morphometric traits are further used to quantify the morphological resemblance between CT scans of corals from field experiments (Fig. 1) [25], [32] and simulated corals.

**Figure 5:**
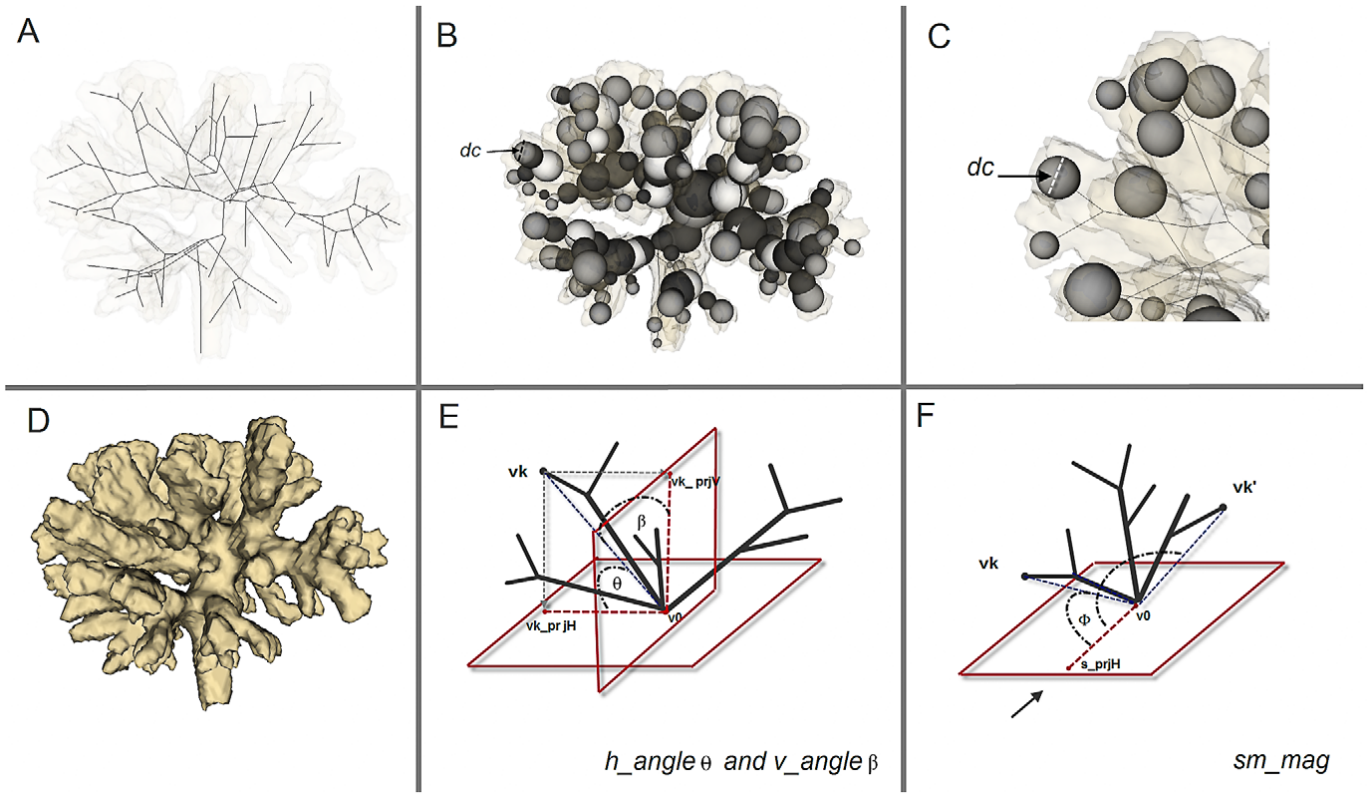
Morphometric methods. (A) A skeleton graph with the increased level of occlusion of the volumetric data in the background, (B–C) Visualization of spheres used for calculating morphometric traits - diameter of a sphere at the terminal branch is defined as terminal branch thickness –*dc*, (D) A visualization of the volumetric data of TS_002 coral (See Table 1 for label), (E) Visualization of symmetry angles *h_angle_* 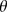 and *v_angle_* 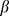, (F) Visualization of the associated vectors used for calculation of symmetry vector *sm_mag_*.

An important pre-processing step of the morphometric analysis is the construction of a morphological skeleton of a 3D object, shown in figure 5A. Skeletonization reduces the object to a network of thin lines, one pixel or voxel thick, running through the centers of the object. A morphological skeleton or medial axis has the same topology (a similar branching structure) as the original object, and occupies the same spatial extent in the image. We used the skeletonization algorithm previously described [35]. The diameter *dc* of the sphere *c* located at the endpoints of the morphological skeleton was determined and represents the local thickness of the terminal branches (see Figure 5B–C). In addition, global geometric properties from simulated and CT-scans corals were extracted: surface, volume and surface/volume ratio estimations (Table 1).

To quantify the symmetry of branch formation in corals and simulated objects, we introduce two extra morphometric variables - the symmetry angles *h_angle_* and *v_angle_* (Figure 5E), and the symmetry magnitude *sm_mag_* (Figure 5F). Let us consider a morphological skeleton graph involves *N* distinct vertices 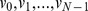, where 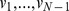 are vertices connected to root node 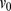 in three-dimensional Euclidean space (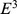). We define a horizontal plane *P_xy_* passing through 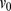 and the orthogonal vertical plane *P_yz_*. The projection of *N-1* vertices on *P_xy_* and *P_yz_* consists of 2(N−1) projected points 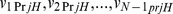 *and* 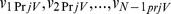 respectively. For each vertex 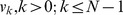, the angles between 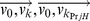 and 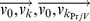 are defined as *h_angle_* and *v_angle_* (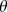 and 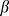 in Figure 5E). The two angles give us the orientation of vertex 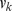 to *P_xy_* and *P_yz_* respectively. Here we define the symmetry angle of the skeleton graph by looking at the distribution of *h_angle_* and *v_angle_*. The morphological skeleton graph is considered to be symmetric (in the horizontal and vertical direction) if the difference between *h_angle_* and *v_angle_* is close to zero i.e. the two angles are the same (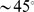).

Furthermore, consider a reference point *s* chosen in the upstream direction of a coral that was exposed to uni-directional flow or *s* as an arbitrary reference point for corals that were exposed to other conditions, 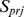 is a projection point of *s* on *P_xy_*. Let 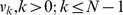 be an arbitrary vertex in a morphological skeleton graph, the scalar projection of vector 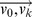 to 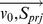 as a magnitude of 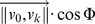, where 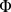 is the angle between 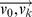 and 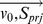. Let us imagine another vertex 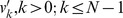 in the skeleton graph that lies exactly in the opposite direction of 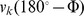, the sum of the two projected vectors (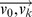 to 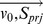 and 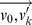 to 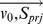) negates each other and thus the vertices 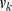 and 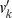 are considered to be symmetric in the direction of the vector 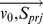 i.e. along streamline direction. The symmetry magnitude *sm_mag_* is defined as 
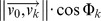
, where 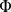 denotes the angle between 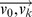 and 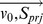. The skeleton graph is considered to be symmetric (along stream line vector 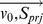) if 
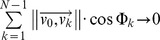
.

The distribution of the symmetry angles *h_angle_* and *v_angle_* gives an overview of branch orientation with respect to flow direction. A skewed distribution of either *h_angle_* or *v_angle_* indicates asymmetrical branching distribution in some direction whereas the sum of *sm_mag_* provides the symmetry of branches along streamline direction.

## Results

### Influence of flow on morphology

**Figure 6:**
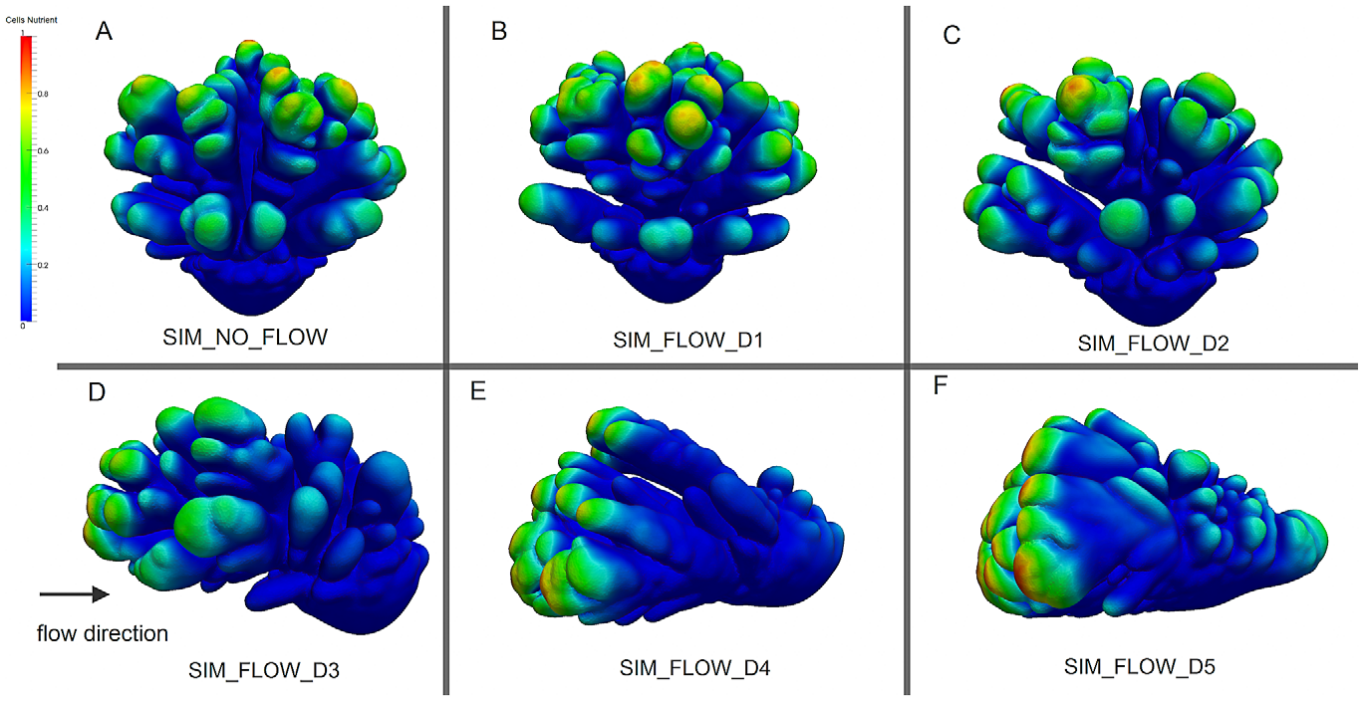
The simulated growth forms. (A) Simulated coral in a no-flow condition. (B–F) Simulated corals from various flow simulations (B) *Pe_branch* = 0.00113, (C) *Pe_branch* = 0.0105, (D) *Pe_branch* = 0.0970, (E) *Pe_branch* = 1.13, (F) *Pe_branch*∼11.3, Arrow indicates flow direction. The labels of the simulated corals are located on the bottom of each figure (See Table 1 for labels).

Under diffusion-limited conditions (no-flow) in the simulations, branches emerge in all directions leading to a relatively symmetrical shape (Figure 6A).

In the subsequent simulations we increased the impact of flow over diffusion by gradually lowering the diffusion coefficient (*D* in Equation 3). We observe that the branches asymmetry increase as the *Pe* number becomes larger. Branches tend to form in the stream upward direction, while they are gradually suppressed in the downstream sides is (Figure 6D–F).

**Figure 7:**
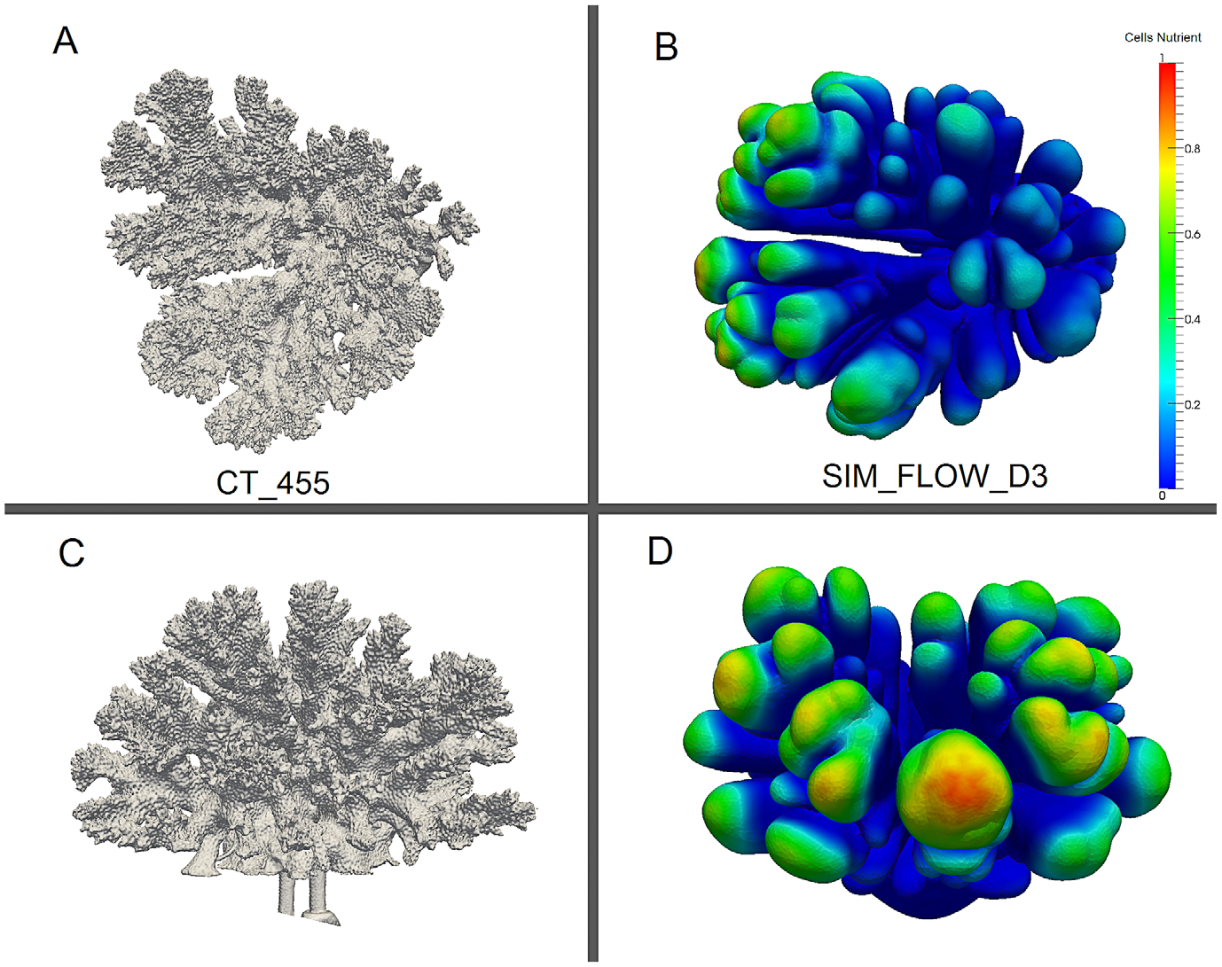
Qualitative comparison between the real (A, C) and simulated coral (B, D) (CT_455 and SIM_FLOW_D3, see **Table 1**).

Similarly to the the coral from the flume tank experiment (Figure 7), an asymmetrical simulated growth form emerges, particularly at a higher *Pe* value, with branches mainly formed on the upstream side (*Pe_branch* = 1.13, Figure 6D and 7).

**Figure 8:**
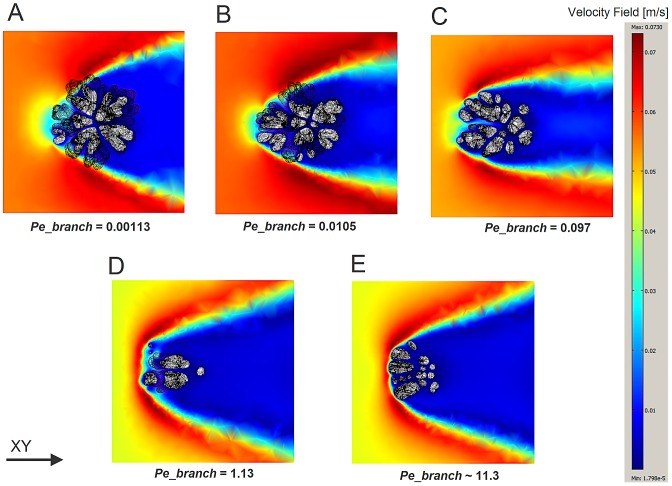
*z*-plane Slices (*z* = 0.07) of the flow pattern around the simulated corals, the slices were taken from the middle part of the colony. (A) SIM_FLOW_D1, *Pe_branch* = 0.00113, (B) SIM_FLOW_D2, *Pe_branch* = 0.0105 (C) SIM_FLOW_D3, *Pe_branch* = 0.097 (D) SIM_FLOW_D4, *Pe_branch* = 1.13 (E) SIM_FLOW_D5, *Pe_branch*∼11.3. Slices of a different z-plane can be found in Supplementary Figure S3 (See Table 1 for the labels).

Considering the flow patterns around the simulated objects, we observe an asymmetrical branching trend with a high degree of compactification with the increasing *Pe* (Figure 8). Flow inside the colony, i.e. between branches, was substantially reduced with most of the flow circumventing the coral's colony (Figure 8D–E). Furthermore it can be observed that the along-stream flow gradient of the simulated object becomes steeper for higher *Pe* numbers leading to a higher degree of absorption of simulated nutrient in the upstream part of the simulated object. The reason for this is because the simulated morphology becomes more compact at a higher *Pe* and hence friction is greater, not because the flow patterns vary with the *Pe*. In other word, the simulated morphology has changed with the increasing *Pe*.

### The influence of flow on surface/volume ratio

To quantify the degree of compactification of the corals and the simulated forms under the influence of different flow conditions, we computed the surface/volume ratio of each form. In general, the surface/volume ratio of *Pocillopora verrucosa* is higher than other species such as *Madracis mirabilis*, *Acropora sp. and Montipora sp.* [35], [37]. A higher surface/volume ratio indicates a higher area which is in contact with the environment and (potentially) a higher amount of mass transfer. The roughness of the coral surface also influences mass transfer rate [16]. Factors that influence the degree of surface roughness include the living tissue and polyps. In reality, it can be observed that surface area will increase when corals extend their polyps thus affecting the mass transfer rate. However, in our study we assume a smooth surface because including all the details about the roughness of a real coral would require 3D images with extremely high resolution, which is currently beyond the capabilities of the available medical scanners.

The surface/volume ratios calculated from the *in situ* corals have a higher value when compared to the surface/volume ratios obtained from the simulations. The controlled coral (CT_456) has the highest surface/volume ratio whereas for the rest of the corals, we found an increasing trend when *Pe_branch* and *Re_branch* increased. However increasing *Pe* number in the simulations tends to causes the surface/volume ratio to decline (Table 1).

### Quantification of the impact of flow on the symmetry of branching growth forms

**Figure 9:**
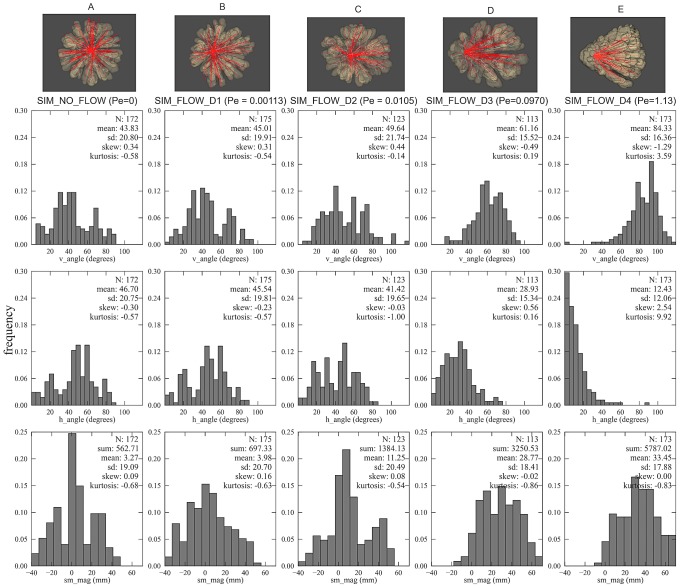
Visualization of the volume rendering of the simulated corals with their associated histograms of the local morphometric traits. Red lines indicate projected branches vector on the substratum plane (visualized from the bottom up perspective). For the simulation with flow, flow is directed from right to left. The morphometric traits measured here are as follow: symmetry angles *h_angle_* and *v_angle_*, and the symmetry attitude *sm_mag_* (see table 1 for labels). (A) SIM_NO_FLOW, *Pe_branch* = 0, (B) SIM_FLOW_D1, *Pe_branch* = 0.00113, (C) SIM_FLOW_D2, *Pe_branch* = 0.0105 (D) SIM_FLOW_D3, *Pe_branch* = 0.097 (E) SIM_FLOW_D4, *Pe_branch* = 1.13.

The degree of symmetry was analyzed by examining the distribution of symmetry angles (*h_angle_* and *v_angle_*) and symmetry magnitude (*sm_mag_*). The mean values (*h_angle_* and *v_angle_*) provide information about the direction of coral's branches while the sum of symmetry magnitude gives the notion of the colony-level symmetry. We first looked at the trend of these two morphometric traits from the simulations and then compared with the CT-scanned corals. For the flow simulations with lower *Pe*, the average values of symmetry angles are 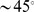 and the sum of the symmetry magnitudes is quite low (Figure 9A and 9B) thus indicating the morphology is symmetric. Under high flow, the symmetry angles diverge from 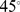 and since they are symmetric to each other the sum of the two angles is always close to 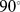 (Figure 9C–9E).

**Figure 10:**
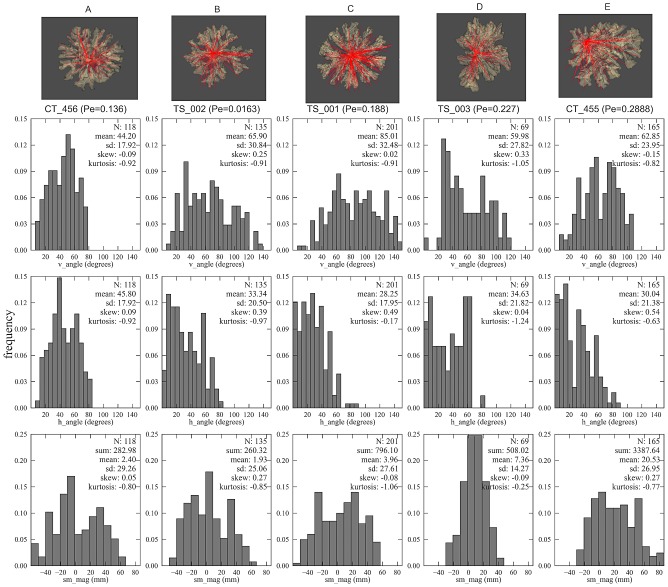
Visualization of the volume rendering of the CT-scanned corals with their associated histograms of the local morphometric traits. Red lines indicate projected branches vector on the substratum plane (visualized from the bottom up perspective). For the *in situ* flow-controlled corals, flow direction is from right to left. The morphometric traits measured here are as follow: symmetry angles *h_angle_* and *v_angle_*, and the symmetry attitude *sm_mag_* (see table 1 for labels). (A) controlled coral CT_456, *Pe_branch* = 0.136, (B) reduced flow coral (TS_002), *Pe_branch* = 0.0163 (C), enhanced-flow coral TS_001, *Pe_branch* = 0.188, (D) enhanced-flow coral TS_003, *Pe_branch* = 0.227, and (E) enhanced-flow coral CT455, *Pe_branch* = 0.288.

We verify the same trend in the CT-scanned corals, for which the sums of the symmetry magnitudes are small for a highly symmetric growth form, but there is a distinction in symmetry angles between the controlled coral and the *in situ* corals. Although the controlled coral (CT_456) exhibits a low sum of symmetry magnitude *sm_mag_* and the symmetry angles are close to 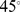 (both *h_angle_* and *v_angle_*), this does not hold true for the *in situ* corals (TS_001 and TS_002, see Figure 10A and 10B) since their symmetry angles diverges from 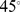 even though they still appear to be symmetric (because of the low *sm_mag_*). This suggests the relationship between the *Pe* number and the associated symmetry angles and magnitudes; that is increasing *Pe* tends to make the sum of the symmetry magnitude higher and induces the symmetry angles to diverge from 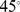 (Figure 10C–10E).

The simulation approach provides an indication of how the simulated corals can change their degree of symmetry by the increasing *Pe*. Under the influence of uni-directional flow, branches form towards the direction of the incoming flow thus reducing the formation of branches on the downstream side. This phenomenon can also be observed in the *in situ* CT-scanned corals.

## Discussion

**Figure 11:**
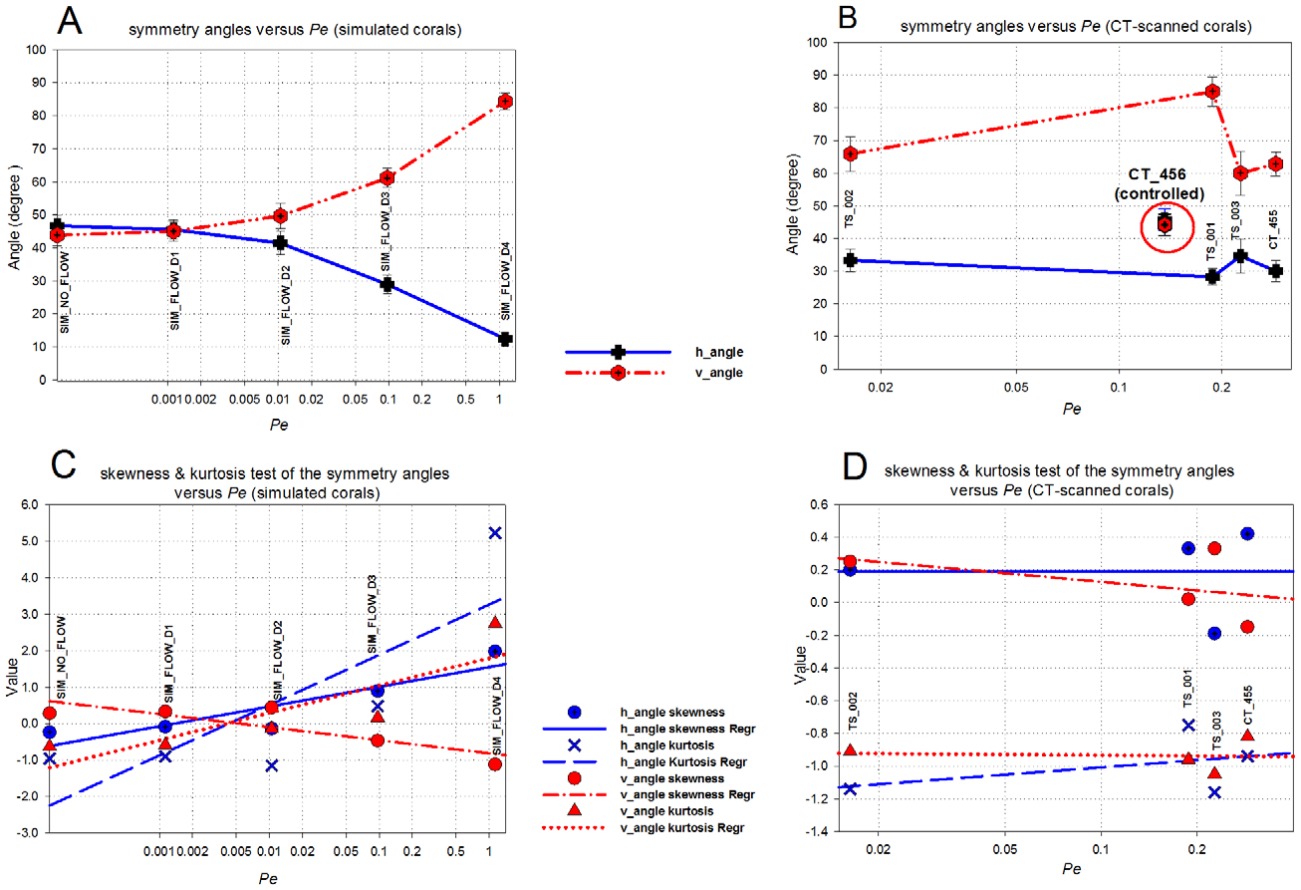
Mean values of symmetry angles *h_angle_* and *v_angle_* versus *Pe_branch* for simulated corals (A) and CT-scanned corals (B). Error bars indicate 95% confidence interval. (C–D) shows regression plot of skewness and kurtosis against *Pe_branch* for both the simulated and CT-scanned corals.

**Figure 12:**
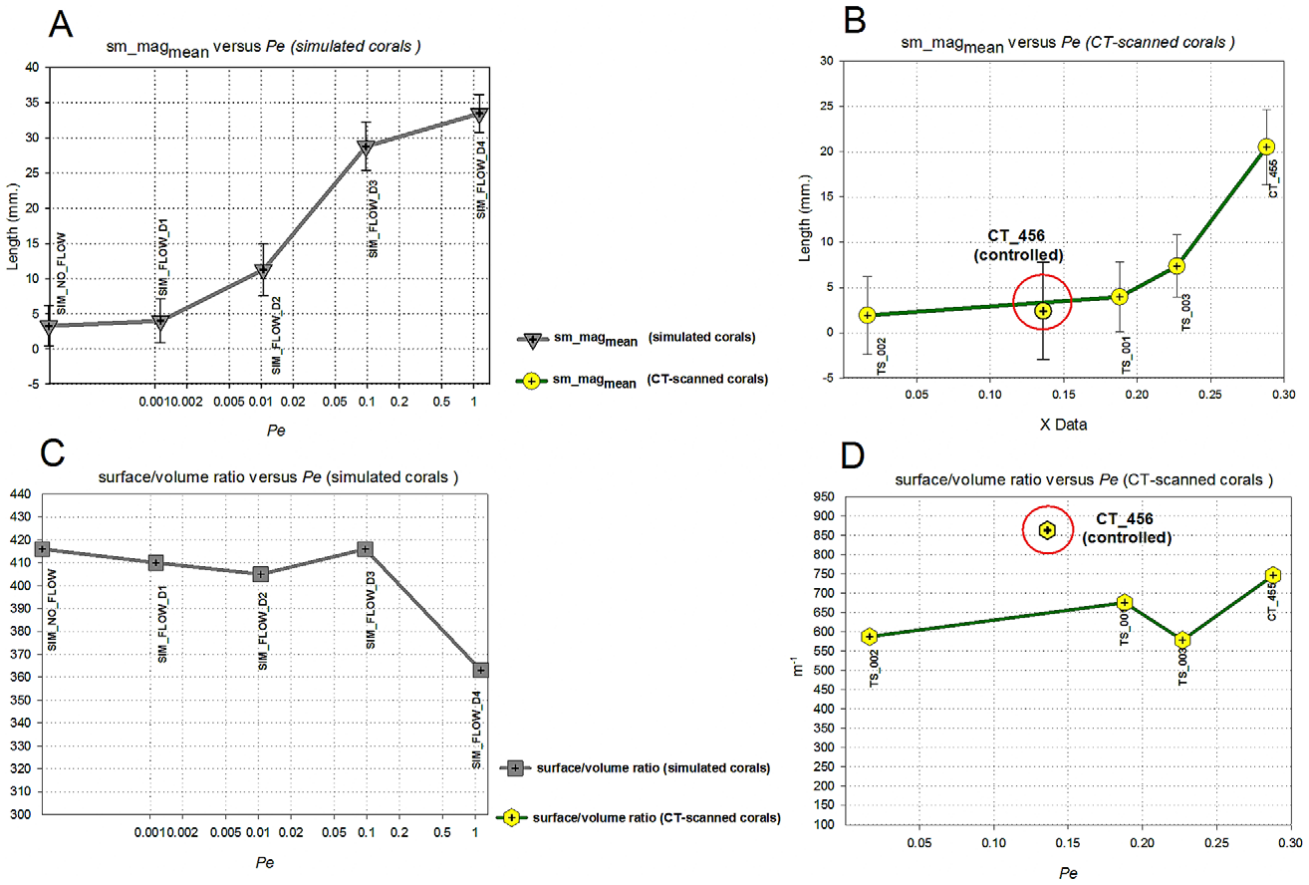
Mean values of symmetry magnitude *sm_mag_* versus *Pe_branch* for simulated corals (A) and CT-scanned (B) corals. Error bars indicate 95% confidence interval. (C-D) surface/volume ratio of simulated and CT-scanned corals versus *Pe_branch*.

Using a simulation approach, we studied the impact of hydrodynamics on the growth of the scleractinian coral *Pocillopora verrucosa*. The 3D morphometric analysis provided a quantitative approach and enabled us to investigate the impact of uni-directional flow. We observed that hydrodynamics plays a major role in the simulated morphologies whereas an increase in *Pe* number induces the formation of asymmetrical branching growth forms (Figure 6). Branches tend to develop in the direction of the incoming current, resulting in an asymmetrical form. In the flow simulations, we found a decreasing trend of the horizontal symmetry angle (*h_angle_*) when increasing *Pe*, as opposed to the vertical symmetry angle (*v_angle_*, see Figure 11A) and the symmetry magnitude (*sm_mag_*, see Figure 12A). A similar trend was found in the CT-scanned corals from the *in situ* experiment (Figure 11B and 12B). According to our analysis, corals seem to maintain their symmetry angles about 45° to the substratum at lower *Pe*. In the simulation these angles will change drastically if high *Pe* number is used. While the simulated corals exhibit a high degree of plasticity, *in situ* corals tend to maintain their symmetry to some extent. For low *Pe* number their symmetry angles were less than 45° but they were observed to be very symmetric, which implies plasticity due to the impact of uni-directional flow.

Our simulation model predicts a decreasing surface/volume ratio when *Pe* increases (Figure 12C) which also occurs in many marine sessile organisms (e.g. sponges, hydro corals, scleractinian corals [13]). In contrast, this trend does not agree with the *in situ* CT-scanned corals (Figure 12D), since they were grown in the controlled experiments *in situ*, where flow (uni-directional) was limited. Hence it is impractical to compare them with the earlier measurements, which used samples from different hydrodynamic regimes growing at a different temporal scale.

The surface/volume ratio provides a significant implication of how corals occupy a certain volume without taking into consideration the temporal scale of their growth. After a period of simulation time, objects from advection-dominated simulations occupy less volume and become more compact, reducing their surface/volume ratio. However, if spatial scale of the growth is used to evaluate the surface/volume ratio, at any rate, flow-induced object will exhibit a higher ratio. For example, considering the interim object (growth step 98) of the diffusion-dominated simulation (Figure 6A), the object occupies a volume of 7.71e^−5^ m^3^ and has a surface area of 2.23e^−2^ m^2^ corresponding to the surface/volume ratio of 289 m^−1^. This value is definitely lower than the ratio measured from the advection-dominated simulation (Figure 6D) that has the surface/volume ratio of 416 m^−1^ (volume = 7.74e^−5^ m^3^ and surface area = 3.22e^−2^ m^2^).

Although our simulations provide a reasonable approximation of a coral growth process and various growth forms emerge in response to the varying *Pe* number, we still face a challenge to include a full scale level of detail. Our simulation cannot approximate all the fine details of *Pocillopora verrucosa* especially the small-scale roughness of the branches with many bumps (hence the Latin time *verrucosa*) but we can simulate other coral species with a relatively simple corallite structure quite well (e.g. *Madracis* see [30]). If the exact approximation is needed, we may have to include the role of corallite for species with complex corallite structure - such as *Pocillopora* or *Acropora -* in our model. Another interesting property of branching corals that we cannot simulate is known as anastomosis –the fusion of branches, a phenomenon which is observed in many branching organisms such as scleractinian corals, hydro-corals and sponges [13] that are exposed to high energy regimes of the reef. If we look at the simulated form in Figure 6, the object with highest *Pe* (Figure 6F, *Pe_branch*∼11.3) is packed with a lot of braches. In reality some of these braches can easily be fused. This property is probably important for the study of coral growth in a higher range of *Re* number.

In our model, we also address the relevant importance of the growth function (Equation 6) to the growth rate. The model parameters from Equation 6 can be interpreted as species-specific variables. The implication of this is the possibility to use real data and attribute them to the simulation model. Although the underlying physiological process is highly simplified, the diffusion-limited assumption still plays an important role to the study of coral's growth [38]. Furthermore, based on the surface diffusion and diffusion-limited assumption, our accretive growth simulation is a good candidate to study the so-called “two compartment proton flux model” [39], where a simplified version of calcification can be incorporated into the accretive function to study the translocation of fixed-carbon energy supply from zone of photosynthesis and zone of rapid calcification. Hence, to assess the effectiveness of relevant parameters such as *Pe* number, *Re* number and surface diffusion, to name a few, an extensive sensitivity analysis is required together with a quantitative comparison between the simulated objects and the real corals (see also [30]).

The methods presented in this paper for modeling accretive growth and the impact of hydrodynamics, in combination with a method for the quantitative analysis of three-dimensional complex shape can be applied to a large class of marine sessile organisms (e.g. scleractinian corals, hydro corals, sponges, rhodoliths). Morphological plasticity is a major issue in different fields of marine and coral biology (e.g. ecology, taxonomy, paleontology) with applications in environmental studies (e.g. coral bleaching and ocean acidification [38]). To our knowledge, only few of the existing computational models aim to understand the emergence of growth and form under different hydrodynamic conditions. Most examples are from physics [40], [41], and a few studies about hydrodynamics effect on growth and form of bacteria colonies [42] or bio-films [43]. Thus far, we have overcome the instabilities issue of the earlier version of the accretive growth model [44], [45] and we are able to simulate coral morphologies at a more realistic flow speed (5 cm s^−1^). We varied *Pe* number by lowering diffusion coefficient (*D* in Equation 3) and maintained a constant *Re* number throughout the simulations (*Re_colony*∼100). While this is crucial to avoid numerical instabilities of the Navier-Stokes equations, our results (the simulated forms) could be slightly different if the *Pe* number was changed by varying the velocity rather than the diffusion coefficient. This issue could be overcome by introducing turbulence flow simulation and study the steady state approximation of Reynolds's Average Navier-Stokes equations. With our provided framework, this can be done in the future using CFD package with turbulence flow solver in COMSOL Multiphysics [31].

To date, our coupled accretive growth model is the first example of a computational model of growth form that can be used to generate objects with a high resemblance to biological growth forms under different hydrodynamic conditions. We can compare and quantify our simulated objects and the real corals using three dimensional morphometrics. Our study also shows that the formation of symmetrical branching forms [25]–[27] can be explained with a biomechanical model. In reality most scleractinian corals will not be growing under uni-directional flow but will be exposed to bi-directional current where the flow direction is reversing twice a day because of the tidal movements. This leads to a next question: is a bi-directional current causing the formation of symmetrical branching colonies? This will be a subject of our future research.

## Supporting Information

Figure S1 **Different views of the simulated growth forms.** Arrow indicates flow direction. Left column side view (XZ plane), right column top view (XY plane) (A) Simulated coral with no-flow. (B–F) Simulated growth forms from different flow simulations (B) *Pe_branch* = 0.00113, (C) *Pe_branch* = 0.0105, (D) *Pe_branch* = 0.097, (E) *Pe_branch* = 1.13, (F) *Pe_branch*∼11.3.
(TIF)

Figure S2 **Simulated growth forms using different values for** ***n*** **in the growth function (** **Equation 5** **).** (A) n = 1.0, (B) n = 1.4, (C) n = 2.2, (D) n = 2.4, (E) n = 2.6.
(TIF)

Figure S3 **Different z-plane slices of the flow pattern around the simulated corals (From top to bottom row; z = 0.09, 0.07, 0.05, 0.03).** Column (A) SIM_FLOW_D1, *Pe_branch* = 0.00113, (B) SIM_FLOW_D2, *Pe_branch* = 0.0105 (C) SIM_FLOW_D3, *Pe_branch* = 0.097 (D) SIM_FLOW_D4, *Pe_branch* = 1.13 (E) SIM_FLOW_D5, *Pe_branch*∼11.3 (see table 1 for labels).
(TIF)

Text S1 **This file demonstrates a MATLAB script describing COMSOL routines (v3.5a) and parameter settings that were used in our advection-diffusion simulations for the first growth step.** Note that the script contains a large set of coordinates which were generated by our simulation code. These co-ordinates were used to interpolate the results from COMSOL after the advection-diffusion simulations.
(M) Click here for additional data file.
