# Supplementary figures and images for: Modelling Growth and Form of the Scleractinian Coral Pocillopora verrucosa and the Influence of Hydrodynamics
Source: PLoS Comput Biol. 2013 Jan 10;9(1):e1002849. doi: 10.1371/journal.pcbi.1002849 (PMC3542083; doi:10.1371/journal.pcbi.1002849)

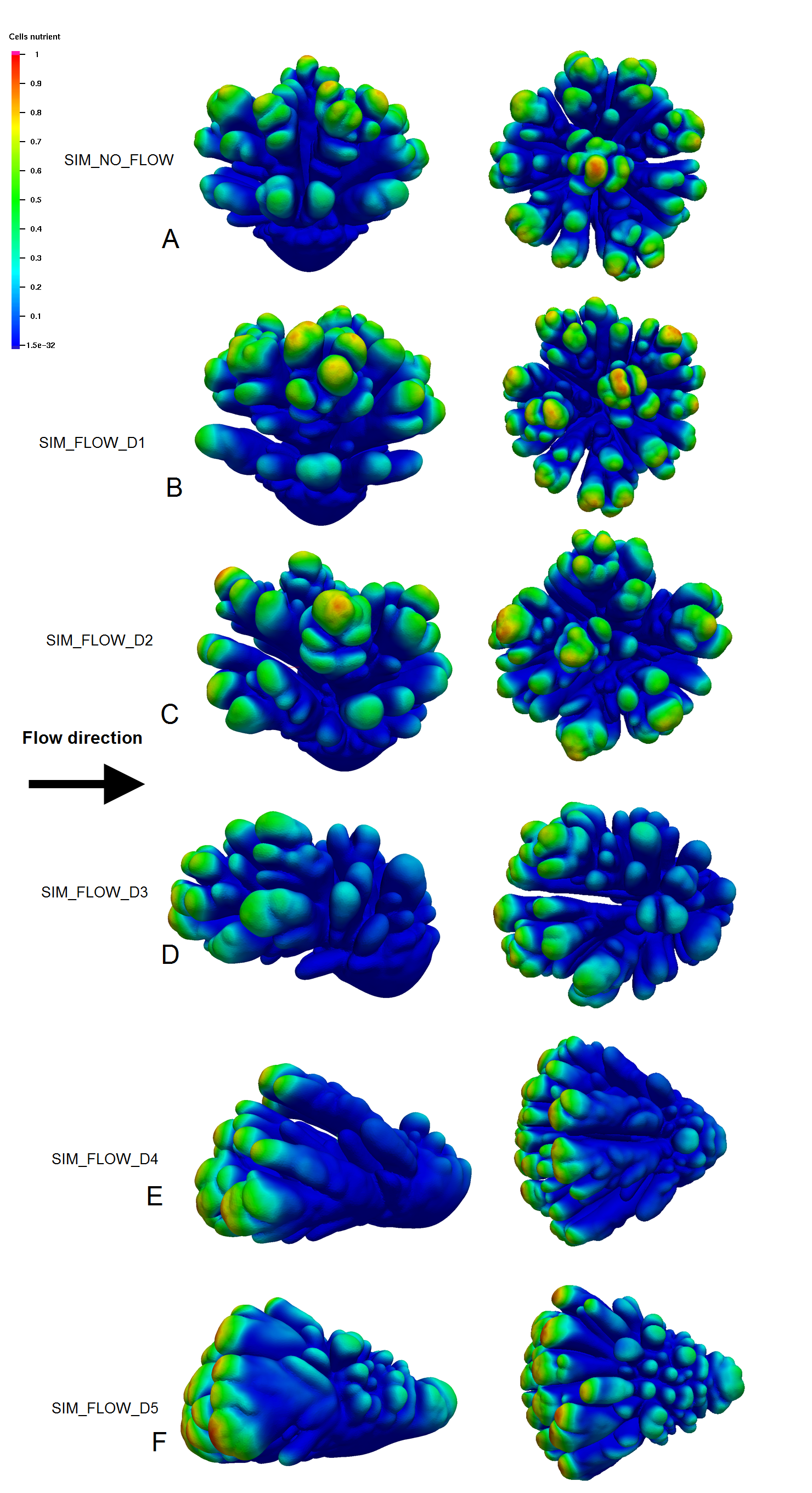

Supplement: Figure S1 — Different views of the simulated growth forms. Arrow indicates flow direction. Left column side view (XZ plane), right column top view (XY plane) (A) Simulated coral with no-flow. (B–F) Simulated growth forms from different flow simulations (B) Pe_branch = 0.00113, (C) Pe_branch = 0.0105, (D) Pe_branch = 0.097, (E) Pe_branch = 1.13, (F) Pe_branch∼11.3. (TIF) [file pcbi.1002849.s001.tif]

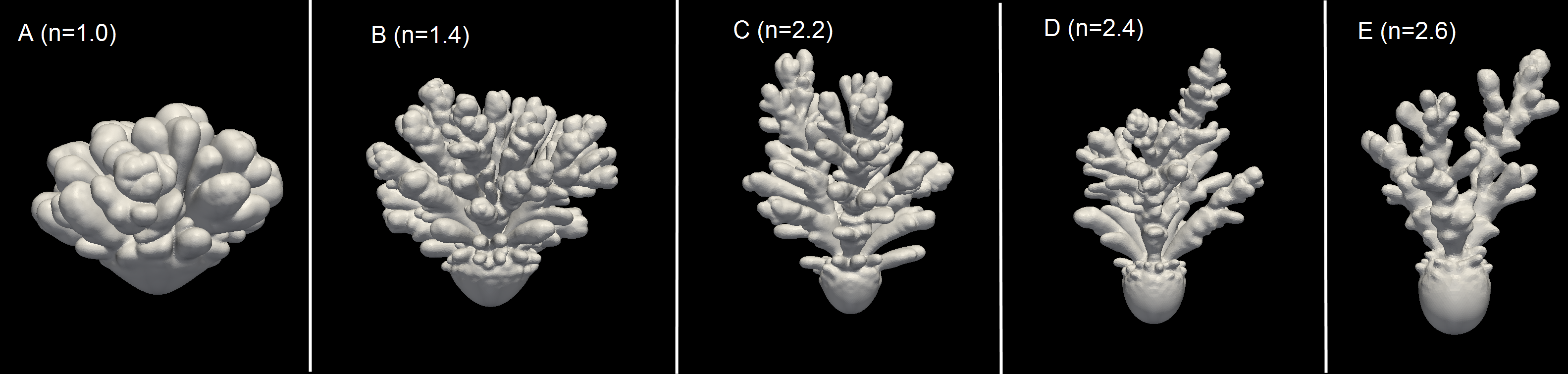

Supplement: Figure S2 — Simulated growth forms using different values for n in the growth function ( Equation 5 ). (A) n = 1.0, (B) n = 1.4, (C) n = 2.2, (D) n = 2.4, (E) n = 2.6. (TIF) [file pcbi.1002849.s002.tif]

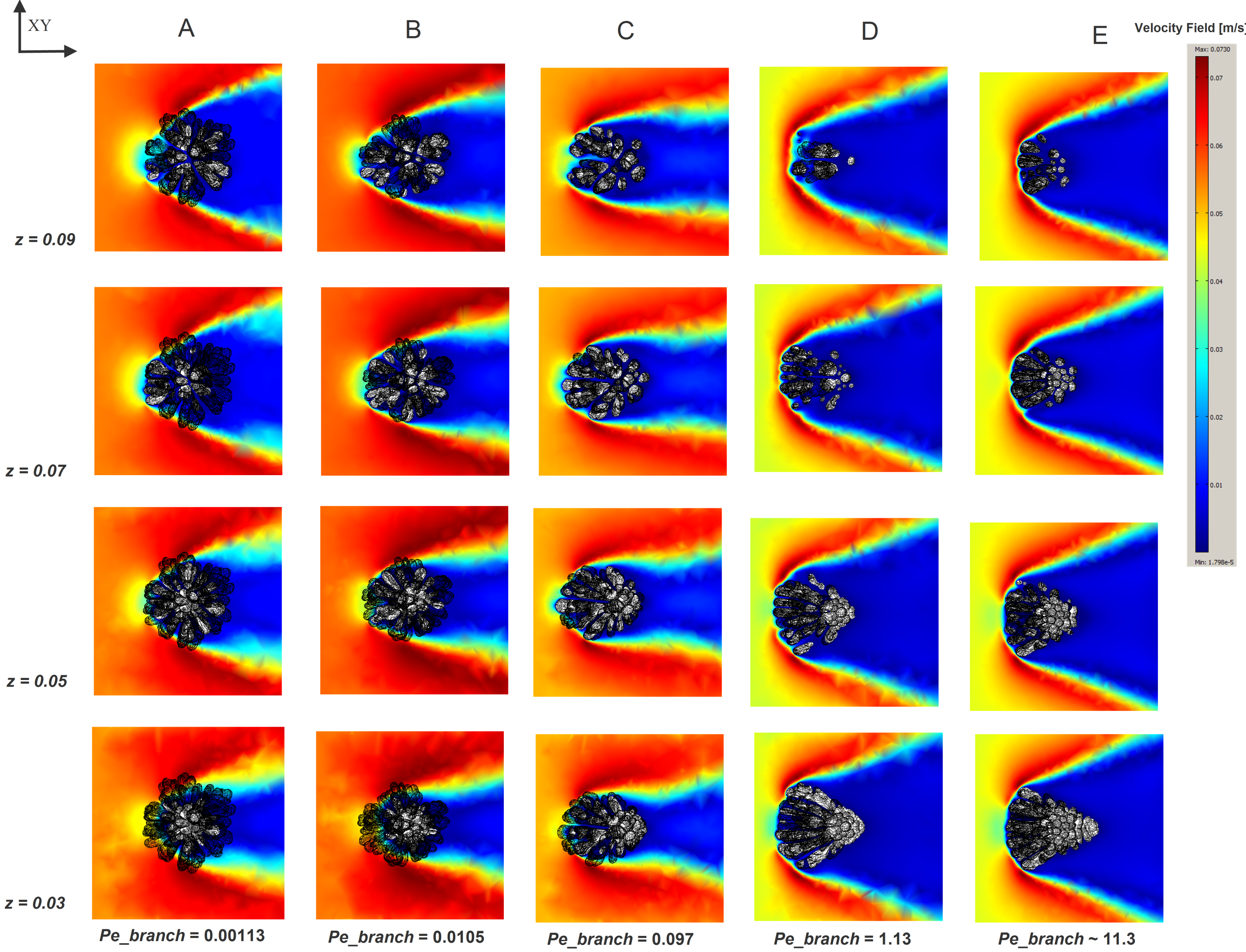

Supplement: Figure S3 — Different z-plane slices of the flow pattern around the simulated corals (From top to bottom row; z = 0.09, 0.07, 0.05, 0.03). Column (A) SIM_FLOW_D1, Pe_branch = 0.00113, (B) SIM_FLOW_D2, Pe_branch = 0.0105 (C) SIM_FLOW_D3, Pe_branch = 0.097 (D) SIM_FLOW_D4, Pe_branch = 1.13 (E) SIM_FLOW_D5, Pe_branch∼11.3 (see table 1 for labels). (TIF) [file pcbi.1002849.s003.tif]
